# Evaluating the safety of a rotavirus vaccine: the REST of the story

**DOI:** 10.1177/1740774508090507

**Published:** 2008

**Authors:** Joseph F Heyse, Barbara J Kuter, Michael J Dallas, Penny Heaton

**Affiliations:** ^a^Merck Research Laboratories, West Point, PA 19486, USA, ^b^Novavax, Rockville, MD 20850, USA

## Abstract

The Rotavirus Efficacy and Safety Trial (REST) was a blinded, placebo-controlled study of the live pentavalent human-bovine vaccine, RotaTeq® (Merck & Co. Inc., West Point, PA). REST was noteworthy because its primary objective was to evaluate the safety of RotaTeq® with regard to intussusception, a rare intestinal illness that occurs with a background incidence of approximately 50 cases per 100 000 infant years. The study involved approximately 70 000 infants at over 500 study sites in 11 countries. The study demonstrated that the risk of intussusception was similar in vaccine and placebo recipients and that the vaccine prevented rotavirus gastroenteritis, ameliorated the severity of disease in those who had any disease, and substantially reduced rotavirus-associated hospitalizations and other health care contacts. This report provides an in-depth review of the background, statistical and regulatory considerations, and execution of REST. We describe the rationale and methods used for sample size, continuous safety monitoring, group sequential design, and detailed study execution. The results of the study have been reported elsewhere. The design and conduct of this study may serve as a useful model for planning other future large-scale clinical trials, especially those evaluating uncommon adverse events.

## Introduction

Rotavirus is the leading cause of hospitalization and death from acute gastroenteritis among infants and young children worldwide [1,2]. In 1998, a tetravalent rhesus-human reassortant rotavirus vaccine (RRV-TV; RotaShield, Wyeth Laboratories) was licensed and recommended by the Advisory Committee for Immunization Practices (ACIP) for routine immunization of infants in the United States [3]. However, postmarketing surveillance studies detected a temporal association between RRV-TV and intestinal intussusception, the telescoping or prolapse of one portion of the bowel into an immediately adjacent segment. Intussusception is an uncommon illness with a background incidence of 18–56 cases per 100 000 infant years during the first year of life in the US [4]. The population attributable risk detected in the postmarketing studies for RRV-TV was approximately 1 additional case per 10 000 vaccine recipients [5–8]. No association between RRV-TV and intussusception was observed in clinical studies conducted prelicensure [9]. Clinical studies did show that a higher proportion of RRV-TV recipients than non-RRV-TV recipients had gastrointestinal illnesses (including vomiting, diarrhea, abdominal pain, and bloody stools), as well as fever [10–13]. RRV-TV was voluntarily withdrawn from the market in October 1999 and 2 weeks later the ACIP rescinded its recommendation for universal vaccination.

At the time the issues arose around intussusception and RRV-TV, clinical development of RotaTeq®, a pentavalent human-bovine reassortant rotavirus vaccine (PRV, Merck & Co., Inc., West Point, PA) was in phase II trials. Various formulations of PRV showed good efficacy (∼70% reduction of episodes of rotavirus gastroenteritis of any severity and 100% reduction of episodes of severe disease) and that the incidence of fever and gastrointestinal symptoms was generally similar among vaccine and placebo recipients [14]. A decision was made to continue the PRV program because of the importance of a safe and effective rotavirus vaccine to public health [15–17]. The absence of an apparent association between wild-type human rotavirus disease and intussusception indicated that intussusception would not necessarily be associated with all rotavirus vaccines [9,18]. Furthermore, preclinical data suggested that there might be some biological differences between PRV and RRV-TV [19, Merck unpublished data]. However, evaluation of the safety of the vaccine with respect to intussusception became a critical question in the clinical development plan, and a large-scale prelicensure safety study was deemed necessary.

The Rotavirus Efficacy and Safety Trial (REST) was a double-blinded (operating under sponsor blinding procedures), placebo-controlled study conducted in 11 countries at over 500 study sites. Study subjects were randomized at a 1 : 1 ratio to receive either the final formulation of PRV or placebo. The study involved approximately 70 000 subjects (including over 35 000 infants (50%) from the United States), making it one of the largest vaccine clinical trials ever conducted prelicensure. The trial demonstrated that the risk of intussusception was similar in recipients of PRV and recipients of placebo. It also demonstrated that PRV prevented rotavirus gastroenteritis and ameliorated the severity of disease in those who had any disease, through the use of a validated scoring system. Furthermore, collection of data on health care encounters among all 70 000 subjects demonstrated that the vaccine substantially reduced rotavirus-associated hospitalization and other health care contacts. The details of the study results have been published elsewhere [20].

This report provides an in-depth review of the statistical, safety, and regulatory considerations involved in the design of the study and the details of the execution of REST. This trial was notable for its design, large sample size, detailed execution, continuous safety monitoring, and lengthy duration. The design and conduct of this study may serve as a useful tool for planning other future clinical trials, especially those evaluating uncommon adverse events.

## Methods

### Key criteria in the design of REST

The primary event of interest in REST was the incidence of intussusception following receipt of PRV or placebo. Intussusception has a clear case definition, and therefore, there was a high level of confidence that if intussusception did occur following vaccination, it could be readily detected. Furthermore, it was known that there were specific time intervals during which the potential risk of intussusception is greatest, both in terms of the age at which intussusception occurs naturally in young infants as well as the time period following RRV-TV when the majority of cases occurred. Thus, a clear case definition and time periods of interest could be easily specified. However, the design of REST faced 3 main challenges: (1) *Study Design Considerations*: To design a study that was large enough to provide a meaningful evaluation of the risk of an uncommon event, intussusception, yet was feasible to implement prelicensure, (2) *Safety Considerations*: To develop a continuous safety monitoring system that would insure a high level of confidence in detecting cases of intussusception and identifying any potential increased risk of intussusception early so as to minimize the risk to trial participants, and (3) *Regulatory Considerations*: To conduct a study that would be acceptable to the regulatory community and provide adequate information for licensure decisions. The method by which each of these challenges was addressed in the design of REST is described in further detail subsequently.

### Study design considerations

The important aspects of the study design included the age at which subjects would be vaccinated, their duration of follow-up, the primary hypothesis, the interim monitoring by the DSMB, the statistical model used, and the sample size. The specifics of each of these aspects are discussed below.

### Age at vaccination

PRV was intended to be used as a 3-dose regimen in infants as part of the routine childhood immunization schedule administered within the first 6 months of life. In this study, it was specifically decided to limit the administration of the first dose of PRV to the period from 6 to 12 weeks of age with subsequent doses at 4- to 10-week intervals. The peak incidence of rotavirus gastroenteritis and associated hospitalizations occurs among infants and young children 6–24 months of age. Thus, it was desired to vaccinate early so as to provide protection at a young age. Completion of the 3-dose regimen within the prespecified time period insured that study participants received all three doses prior to the age of greatest risk of rotavirus gastroenteritis.

This time period is also prior to the period when the background rate of intussusception increases substantially. The background rate of naturally occurring intussusception is fairly low in infants <4 months of age (50 per 100 000 person years), with the rate increasing with age [9]. Restriction of vaccination to the prespecified time period allowed for rapid identification of a possible increase in intussusception with vaccination, should one exist. If intussusception cases were observed in infants at this young age when the background rates are low, it would have been a signal of a possible increased risk of intussusception which would have led to stopping the study early.

### Duration of follow-up

The duration of safety follow-up after administration of each dose of vaccine/placebo was established as 42 days because this time period has traditionally been used for observation of adverse events following live virus vaccines. It was also chosen because it was possible that the cases of intussusception following PRV might occur within a different time frame than those observed after vaccination with RRV-TV. Although the majority of cases of intussusception following RRV-TV occurred within the first 7 days after vaccination, it was possible that because PRV was less reactogenic and only elicited a minimal amount of vaccine viral replications in the intestinal tract, cases of intussusception might be seen later than those following RRV-TV. However, because the risk of intussusception associated with RRV-TV was highest during the week after vaccination, the incidence of intussusception following PRV was also evaluated for the 7 days after vaccination. For long-term evaluation of intussusception, all subjects were to be followed for 1 year after the first vaccination, or until the end of the closure of the study site, whichever occurred first.

### Primary hypothesis and utilization of group sequential design

The primary safety objective of the study was to demonstrate with a high level of confidence that the vaccine had a clinically acceptable safety profile for licensure. This would be shown analytically with the lack of a statistically increased risk and also a low level of risk within the period following vaccination when the vaccine is biologically active. Safety endpoints other than intussusception were also collected and analyzed thoroughly, but the study design was driven primarily by intussusception. The primary safety hypothesis was that PRV would not increase the risk of intussusception relative to placebo within 42 days after any dose. In order to satisfy the primary safety hypothesis, two criteria needed to be met:

1. Throughout the study, the vaccine/placebo case ratio could not reach predefined unsafe boundaries being monitored by the Data Safety Monitoring Board (DSMB) within 42 days following any dose or within 7 days following any dose.
2. At the end of the study, the upper bound of the 95% confidence interval (CI) estimate of the relative risk of intussusception was ≤10 within 42 days following any dose.

These criteria were developed by the sponsor and were part of the study protocol. They were supported by the DSMB for use as their guidelines.

It is well understood by clinical trial statisticians that a single study can never prove the absence of risk. This is especially the case when evaluating uncommon adverse events like intussusception, as in REST. The study design used an end-of-study criterion threshold of risk based on the expected low background rate of intussusception. The success of the study was based on simultaneously satisfying the two criteria listed above. For the end-of-study criterion, the study employed a group sequential design, which called for a minimum of 60 000 infants with complete safety follow-up, and subsequent groups of 10 000 infants if the end of study criterion was not met, up to a maximum sample size of 100 000. These design factors are discussed more fully later in this report.

For the interim safety monitoring, the boundary points were chosen to detect a statistically significant increase in relative risk. In other words, the boundary points corresponded to the lower bound of the 95% CI for relative risk of intussusception among vaccine as compared with placebo recipients being >1.0 at any point in the study; these boundary points were intentionally chosen without regard to multiplicity in order to be conservative. If at any time during the study this boundary was reached for the 7-day or 42-day periods after any of three doses, the DSMB could recommend stopping the study due to safety concerns.

The use of 10 as the upper bound criterion at the end of the study was chosen due to the relatively few intussusception cases expected in the study. Given the number of cases expected, vaccine/placebo risk ratios that were considered clinically acceptable were defined statistically as those relative risk estimates having an upper bound of the 95% confidence interval ≤10. The study was planned assuming a background incidence of 50 cases per 100 000 person years and an exposure window of 102 days, allowing for 30 days post dose 1, 30 days post dose 2, and 42 days post dose 3. (A conservative 30 day post doses 1 and 2 time period was used in planning the study to accommodate the vaccine schedules for some of the countries participating in the study.) For a provisional starting sample size of 60 000, the expected number of total intussusception cases occurring during the 42-day period following any dose of vaccine/placebo was 8, assuming that the vaccine did not increase the risk of intussusception. With 8 observed cases, a case split of 4 vaccine to 4 placebo cases (relative risk of 1) or more favorable was needed to satisfy the primary safety hypothesis at the end of the study. With 10–12 observed cases, relative risks of ≤2 were needed in order for the upper bound to be ≤10. Therefore, only a small relative risk would allow for a conclusion that the vaccine had an acceptable safety profile.

### Statistical model

The basic statistical model for the study assumed that numbers of cases of intussusception were distributed as Poisson random variables with rate parameters λ_V_ and λ_C_ for vaccine (V) and placebo (C) recipients, respectively. Then, conditional on the total number of intussusception cases, the number of vaccine cases is distributed as a binomial with parameters *t* = V + C and π = λ_V_/(λ_V_ + λ_C_). Using this model, the relationship between the relative risk *R* = λ_V_/λ_C_ and π are given by

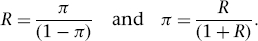


Exact binomial inference was then utilized with *t* and π.

**Figure 1:**
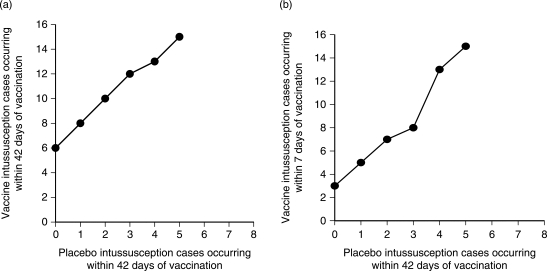
Predefined safety boundaries for the 42 and 7 day ranges after any dose: (a) 42-day period after any dose, (b) 7-day period after any dose

For the interim safety monitoring, the boundary points were chosen to detect a statistically significant increase in relative risk in vaccine compared to placebo recipients. For t total accrued intussusception cases, the boundary point was the smallest number of vaccine cases *v* such that 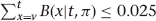, where *B*(*x*|*t*, π) represents the probability of observing *x* vaccine cases among *t* assuming a binomial proportion π. The day 1–42 boundary, shown in Figure 1(a), corresponds to π = 0.5. The day 1–7 boundary compared the number of vaccine cases occurring 1–7 days after any dose to the number of placebo cases occurring 1–42 days after any dose; the day range for the placebo cases remained 1–42 in order to increase the power of this safety monitoring through use of more potential cases. Thus, Figure 1(b) was computed for this scenario using π = 21/102 = 0.171 to account for the differential exposure time in vaccine and placebo recipients.

**Figure 2:**
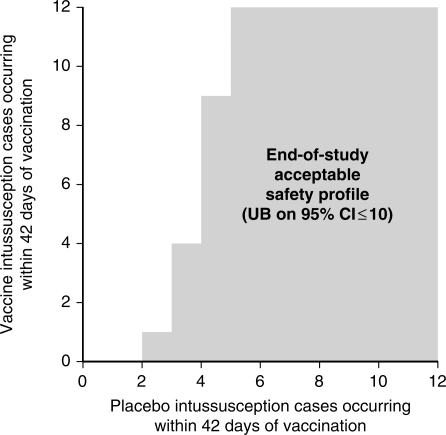
Criteria for stopping enrollment (based on primary hypothesis)

The acceptable safety profile for stopping enrollment in accordance with the group sequential design was based on satisfying the upper bound of the 95% confidence interval estimate of relative risk being ≤10. The shaded region in Figure 2 shows case splits meeting this criterion. As previously discussed, the vaccine/placebo case splits that make up this region represent estimates of relative risk that also would be considered clinically acceptable for licensure. This region was also determined using exact binomial methods, with the impact of the interim stopping rules for negative results being considered. Because the study used interim stopping rules for negative results that were appropriately strict (and stricter than rules designed just to accept the primary null hypothesis) and that covered two different day ranges (1–7 and 1–42), the group-sequential region of acceptance could not be determined using standard design techniques. Instead, the region was chosen according to that defined by the maximum number of vaccine cases *v* such that 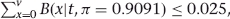, corresponding to a relative risk of 10, and then evaluation of the design properties was done via simulation (described below) in order to account for the interim monitoring. The terminal *P*-value, point estimate, and confidence interval estimate of the relative risk were appropriately adjusted based on the group-sequential aspect of the design using the methodology described in Jennison and Turnbull [21].

**Figure 3:**
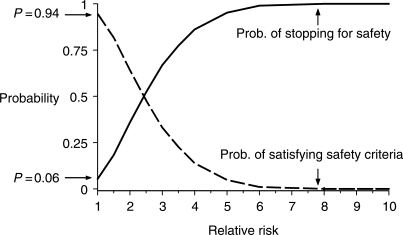
Statistical operating characteristics for REST study design

The REST study design had two simultaneous goals: (1) A high probability that if the vaccine was associated with an increased risk of intussusception this would be detected quickly and the study would be stopped early, and (2) A high probability that a safe vaccine would meet the end of study criteria. The statistical operating characteristics of REST were estimated using Monte Carlo simulation. Each simulation run generated 10 000 random sequences of vaccine and placebo cases. For each sequence, it was determined whether the sequence caused the study to (1) stop early according to the two safety monitoring boundaries, or (2) satisfy the primary safety criteria for stopping the study. Figure 3 shows the probability of each possible outcome for different levels of relative risk. For a vaccine with no increased risk of intussusception, there was a 0.06 probability that the study would stop early due to a safety concern, and a 0.94 probability of successfully reaching the end-of-study criterion. Figure 3 also shows the important role of the continuous safety monitoring in drawing a study conclusion. The probability of stopping the study early for safety concerns increased substantially for relative risks in the range of 2.5–6. For relative risks of 6 or greater, the study would have ended early almost with certainty. The simulation also allowed varying the relative risk profile over intervals of time through the follow-up period. This feature of the simulation was important in that it allowed modeling the risk profile reported for the RRV-TV in the CDC case-control and case series studies [7]. The same five intervals (1–2 days, 3–7 days, 8–14 days, 15–21 days, and 22–42 days) following each of the three doses were used. The probability of reaching one of the two unsafe boundaries was 0.85 for the CDC case-control profile and 0.91 for the CDC case-series profile.

### Safety considerations

Three elements were critical to the safety surveillance used in this study: (1) Active and passive surveillance to insure detection of cases of intussusception, (2) Extensive interim safety monitoring, and (3) Early termination of the study, if deemed necessary. Although not a formal part of the safety monitoring system established to address intussusception, another consideration was the ability to generate adequate general safety data to support licensure at the end of the study.

### Active and passive safety surveillance

In order to evaluate the primary safety objective, all subjects in the study were to be followed for intussusception for a minimum of 42 days following any vaccination. Active follow-up was employed throughout the 4-year study to insure a high level of detecting cases of intussusception. The parent/legal guardian was instructed to report any potential case of intussusception that occurred at any time to the study site. Study sites were required to implement a rigorous program of active safety follow-up for intussusception. This included telephone contacts or home visits on Days 7, 14, and 42 following vaccination to ask about potential cases of intussusception or any other serious adverse experience. If a case of intussusception was reported to the study site, the study site was requested to report the case within 24 h to the sponsor.

Additional safety surveillance was conducted every 6 weeks from day 43 after dose 3 to day 365 after dose 1 or until the study sites end-of-study date, whichever came first. This surveillance was conducted in order to assess any potential cases of intussusception that may have occurred in the year after dose 1. The parent/legal guardian was contacted every 6 weeks by an electronic or postal mailing, telephone call, home visit, or during a physician's office visit and asked about potential cases of intussusception.

Rigorous safety surveillance throughout this 4-year study was critical. Study sites were chosen on the basis of a good standard of care for the treatment of intussusception and the ability to conduct extensive surveillance rather than on the ability to enroll large numbers of infants. In order to assure that study personnel performed all planned safety contacts throughout the study, centralized computer or manual tracking systems were initiated in every country. These systems were developed so that the study sites were reminded when the day 7, day 14, and day 42 contacts, and subsequent 6 week contacts were scheduled for each subject. If contacts were not made within a prespecified timeframe (∼1 day for the Day 7, 14, and 42 contacts and ∼2 weeks for the 6 week contacts), then the study personnel received a reminder notice. It was also important that subjects were not lost to follow-up. At a minimum, safety follow-up was needed for 42 days after any vaccination. Throughout the study, detailed instructions were provided to study site personnel on how to pursue subjects who were potentially lost to follow-up including additional phone calls, mailing of registered letters, and home visits, as appropriate. If a subject was truly lost to follow-up, the sponsor was contacted within 24 h. This rigorous active surveillance system ensured that the data on potential intussusception cases and other serious adverse experiences were as complete as possible. Using these multiple follow-up methods, safety follow-up was completed for >99.9% of subjects enrolled in the study.

In addition to the active surveillance that was performed, a passive surveillance system was developed for all parents/guardians. All parents/guardians were provided with the definition of gastroenteritis (a stomach illness with diarrhea and/or vomiting) and the signs/symptoms of intussusception. Each parent/guardian was asked to notify study personnel if a subject had symptoms compatible with intussusception or was admitted to the hospital or seen in the emergency room for gastroenteritis. Each parent also received a study card that could be used to notify health care personnel that a subject was participating in this trial. The card instructed the health care professional in how to contact study personnel should their patient have any illness that might be intussusception. All illnesses that were reported in which the diagnosis of intussusception was considered, even if the final diagnosis was not intussusception, were assessed.

#### Interim safety monitoring by SEAC and DSMB

A blinded Safety Endpoint Adjudication Committee (SEAC) reviewed each potential case of intussusception when it was reported. A standard operating procedure, which included definitive clinical, radiographic, surgical, and pathology guidelines for the diagnosis of intussusception, was used by the SEAC when adjudicating each potential case of intussusception. The positively adjudicated cases meeting the predefined case definition were then forwarded to a separate, independent Data Safety Monitoring Board (DSMB).

The DSMB was instituted in order to detect a potential increased risk of intussusception early and minimize the risk to trial participants if the risk of intussusception was deemed unacceptable. The DSMB unblinded the treatment group for each positively adjudicated intussusception case when it was reported and made recommendations regarding study continuation after each case. The DSMB plotted the occurrence of each case of intussusception on the graphs shown in Figure 1(a) and (b) for the 7- and 42-day timeframes following vaccination, respectively. The DSMB then utilized the predetermined stopping boundaries in these figures to determine whether there was a statistically significant increase (lower bound of the 95% CI > 1.0) in intussusception risk among vaccine as compared to placebo recipients. These criteria were used in concert with clinical judgment when making interim recommendations regarding study continuation.

The DSMB also met every 6 months over the course of the 4-year study to review all safety data to determine if there were any other adverse events of concern.

Finally, the DSMB made recommendations to the sponsor regarding the completion of the overall enrollment with respect to the group sequential design based on whether the criterion for stopping enrollment associated with the primary safety hypothesis had been satisfied (Figure 2).

### Regulatory considerations

The design of REST was discussed with several regulatory agencies prior to study start. In the United States, the design of the study was formally presented to the Vaccines & Related Biologics Product Advisory Committee (VRBPAC) in May 2000. At this meeting, it was incumbent on the sponsor to propose specific criteria to demonstrate the safety of the vaccine for licensure. Those criteria (described earlier in this document) were unanimously approved by VRBPAC along with the study design. VRBPAC also requested that revisions be made to the consent form for the study to adequately inform parents/guardians of the safety issues being evaluated among study participants.

Numerous discussions were held with the Centers for Biologics Evaluation & Research (CBER) prior to study start in January 2001. The regulatory authorities requested a full review of all prior safety data for the vaccine. They also asked the sponsor to demonstrate a high level of confidence that the vaccine was safe while minimizing the risk to participants in the REST trial. In addition to the standard collection of data on serious adverse experiences from all subjects, the sponsor was asked to solicit detailed safety data on 5000–10 000 susceptible infants gathered from blinded, placebo-controlled studies. The safety criteria for the vaccine were discussed in-depth at a workshop convened by FDA, the National Institutes of Health (NIH), and the Centers for Disease Control and Prevention (CDC) in November 2001 to evaluate the safety of new vaccines [22]. All of this regulatory input was addressed in the design of the study.

## Discussion and Conclusions

REST utilized a unique study design. Since it was necessary to both detect an elevated risk of intussusception at an early stage and to demonstrate that the safety profile of the vaccine was acceptable for licensure, a novel study design was necessary. Specifically, a continuous monitoring boundary was used in conjunction with a group-sequential design. The monitoring boundary was designed to detect an elevated risk in intussusception during biologically and clinically important timeframes while the group sequential design, with its large sample size and possibility of expanding enrollment to accrue more cases of intussusception, allowed for the demonstration of safety at the end of the study. Both the monitoring boundary and the group sequential design were critical to the demonstration of an acceptable product safety profile to support licensure and acceptance in the medical community.

Group-sequential designs had not been routinely used in clinical trials of new drugs or vaccines at the time REST was designed. This type of design has its pros and cons. The advantage of using this design is that it has the potential to draw a conclusion based on a more efficient sample size. The initial sample size for REST was 60 000 subjects, but an additional 10 000 subjects had to be enrolled because the criteria for stopping enrollment according to the primary endpoint was not met based on 60 000 subjects. Had this study been conducted using a classical fixed subject statistical design, the sample size would have been ∼85 000 subjects.

The disadvantage of the group sequential design is that the actual duration of the study and the study budget cannot be predetermined, resulting in the need to work within a range of study completion dates and a range of possible study costs. Uncertainty regarding the timing of study results, let alone the results themselves, can have a major impact on key decisions in moving a product forward, particularly with respect to investing in new manufacturing facilities and the need to develop backup compounds.

The detailed analysis of the association between intussusception and RRV-TV conducted prior to the start of this study necessitated the successful design of REST. Based on the previous experience with RRV-TV, we designed a study of PRV in which the age and timing at which intussusception occurred with RRV-TV was covered. Had the prelicensure studies of RRV-TV been conducted using a similar design, it is likely (≥85%) the studies of RRV-TV would have been stopped due to a safety concern, based on the safety boundaries, as was shown by the simulation results.

The success of this study was possible not only because of its large sample size but also because of the excruciating attention to detail employed in the study's execution. Extensive systems were utilized to track each subject in the trial. The success of these systems was shown by the remarkably low rate of subjects lost to follow-up (<0.1%). A low loss to follow-up rate is particularly important when trying to evaluate an uncommon serious adverse event such as intussusception. Furthermore, the reporting of potential cases of intussusception was handled in a conservative manner so that it was highly unlikely that a case could be missed. These methods, in conjunction with the routine involvement of the SEAC and DSMB in the evaluation of every possible case of intussusception, resulted in a very complete safety monitoring system.

A basic question in evaluating any new drug or vaccine is how much safety data are enough. REST was one of the largest prelicensure studies ever conducted for a vaccine. The prelicensure safety of most new vaccines is based on a sample size of ∼5 000–10 000 subjects. The fact that this study was designed to address a specific concern over the relationship of PRV and intussusception and that no relationship was identified when comparing ∼35 000 vaccine recipients to ∼35 000 placebo recipients provides substantial reassurance to health care professionals, parents, and regulatory agencies of the safety of this vaccine. Nevertheless, postlicensure safety studies of PRV were still requested by regulatory agencies to gather additional information on the safety of the vaccine due to possible differences in the characteristics of infants who receive the vaccine in routine use compared with infants in clinical trials. Two controlled studies are now ongoing with this vaccine, one in a health maintenance organization conducted by the manufacturer involving approximately 44 000 subjects and one using the Vaccine Safety Datalink (VSD) cosponsored by FDA and CDC involving approximately 90 000 subjects. Both studies use a continuous monitoring of intussusception cases as they accrue with a pre-established safety boundary to alert investigators of any increased risk. In total, the safety data from pre- and postlicensure controlled studies will include over 169 000 recipients of PRV, a remarkably large number. The data obtained to date from the postlicensure studies confirm the excellent safety profile of the vaccine and lack of association with intussusception demonstrated in the prelicensure studies.
